# Advancements in neuroregenerative and neuroprotective therapies for traumatic spinal cord injury

**DOI:** 10.3389/fnins.2024.1372920

**Published:** 2024-05-15

**Authors:** Gregor Fischer, Linda Bättig, Martin N. Stienen, Armin Curt, Michael G. Fehlings, Nader Hejrati

**Affiliations:** ^1^Department of Neurosurgery, Cantonal Hospital St.Gallen, Medical School of St.Gallen, St.Gallen, Switzerland; ^2^Spine Center of Eastern Switzerland, Cantonal Hospital St.Gallen, Medical School of St.Gallen, St.Gallen, Switzerland; ^3^Spinal Cord Injury Center, University Hospital Balgrist, Zurich, Switzerland; ^4^Division of Neurosurgery and Spine Program, Department of Surgery, University of Toronto, Toronto, ON, Canada; ^5^Division of Genetics and Development, Krembil Research Institute, University Health Network, Toronto, ON, Canada

**Keywords:** spinal cord injury, neuroregeneraion, neuroprotection, cell based therapies, biomaterials, neuromodulation

## Abstract

Traumatic spinal cord injuries (SCIs) continue to be a major healthcare concern, with a rising prevalence worldwide. In response to this growing medical challenge, considerable scientific attention has been devoted to developing neuroprotective and neuroregenerative strategies aimed at improving the prognosis and quality of life for individuals with SCIs. This comprehensive review aims to provide an up-to-date and thorough overview of the latest neuroregenerative and neuroprotective therapies currently under investigation. These strategies encompass a multifaceted approach that include neuropharmacological interventions, cell-based therapies, and other promising strategies such as biomaterial scaffolds and neuro-modulation therapies. In addition, the review discusses the importance of acute clinical management, including the role of hemodynamic management as well as timing and technical aspects of surgery as key factors mitigating the secondary injury following SCI. In conclusion, this review underscores the ongoing scientific efforts to enhance patient outcomes and quality of life, focusing on upcoming strategies for the management of traumatic SCI. Each section provides a working knowledge of the fundamental preclinical and patient trials relevant to clinicians while underscoring the pathophysiologic rationale for the therapies.

## Introduction

Traumatic spinal cord injury (tSCI) imposes significant physical, social, and financial burden to individuals and their families. tSCI is characterized by damage to the spinal cord, resulting in temporary or permanent functional alterations. The incidence of tSCI ranges from 10.4 to 83 cases per million per year globally, with over 9,000 new cases in European countries per year (Singh et al., 2014; Scivoletto et al., 2017). Notably, the cervical spinal cord is predominantly implicated in tSCI, leading to severe impairments including quadriplegia, respiratory compromise, and reliance on assistance for daily activities. Confronting a persistent rise in the prevalence of spinal cord injuries, there has been a notable surge in scientific attention toward neuroprotective and neuroregenerative strategies. Over the past five decades, there has been a rise in the proportion of high cervical injuries and a decline in the percentage of low cervical injuries (Chen et al., 2016). The proportion of motor incomplete injuries (AIS C and D) increased from 36.4 to 53.2%, whereas AIS A (i.e., sensorimotor complete) have decreased from 53.8 to 33.7% (Chen et al., 2016). To date, no cure for this condition has been found and only few patients suffering from tSCI experience a full restoration of their lost neurological function (Kucher et al., 2018). Hence, the significance of additional research is underscored by the increasing number of published data in the field of tSCI (Wong et al., 2023).

This review aims to offer a comprehensive summary of the contemporary state-of-the art acute management of tSCI along with an overview on recent and promising neuroprotective and neuroregenerative therapies that are currently under investigation.

### Pathophysiology of SCI

The pathophysiology of tSCI involves a complex and multifaceted processes, typically initiated by a traumatic event, which results in direct physical damage to the spinal cord (Ahuja et al., 2017). This primary injury sets off a cascade of secondary events that can further impair microstructural integrity of the spinal cord, thereby resulting in protracted neurologic decline and mitigate functional recovery.

#### Primary injury

The initial mechanical damage, which can be caused by compression, transection, or contusion of the spinal cord, disrupts the integrity of the spinal cord’s neurons, axons and their respective conductivity, as well as vascular structures and the blood-spinal cord-barrier (Singh et al., 2014; Scivoletto et al., 2017). This damage is instantaneous and irreversible, resulting in immediate neurological deficits and followed by the secondary injury (Ahuja et al., 2017).

#### Secondary injury mechanism

Following the primary injury, a series of biochemical and cellular processes, such as impaired neuronal homeostasis (Fehlings et al., 2017) ensue, exacerbating the initial damage. This phase can last for weeks to months and includes vascular damage, such as hemorrhage and ischemia, which lead to disruption of the BSCB and impaired microcirculation, which deprive neurons of essential nutrients and oxygen. Apoptosis is dysregulated due to a dysbalance of intra- and extracellular calcium (Schanne et al., 1979). An acute inflammatory response is triggered resulting from the activation of the innate immune system, mediated by cytokines and chemokines released by astrocytes, microglia, endothelial cells, and peripheral immune cells (Yates et al., 2019). A number of studies have demonstrated the systemic upregulation of inflammatory cytokines such as IL-1, IL-6, and TNF within hours of tSCI in the blood stream and cerebrospinal fluid (CSF) (Hellenbrand et al., 2021). This upregulation subsequently leads to additional infiltration of macrophages, microglia, and neutrophils (Hellenbrand et al., 2021), which further nurture the inflammatory response by secreting additional cytokines and chemokines, all of which is facilitated due to damage of the BSCB and loss of tight junction proteins (DiSabato et al., 2016). The magnitude of neuroinflammation is influenced by the size of the primary insult with an excessive immune response leading to additional cell death.

Glutamate is released by dying neurons and astrocytes, which causes an overactivation of NMDA (*N*-methyl-d-aspartate), AMPA (α-amino- pro- 3-hydroxy-5-methyl-4-isoxazole propionic acid) and kainite receptors, which in conjunction with the ATP-loss related dysfunction of sodium channels can result in elevated intracellular concentrations of Na + and Ca^2+^ (Vanzulli and Butt, 2015). The increased Ca^2+^ concentration further inhibits mitochondrial respiration, leading to energy depletion and dysfunction of the Na^+^/K^+^ ATPase thereby exacerbating axonal membrane depolarization, causing excessive Na^+^ influx across axonal membranes (Vanzulli and Butt, 2015). The dysregulation of ions then promotes cytotoxic cell edema, the production of reactive oxygen species (ROS), and mitochondrial dysregulation (Vanzulli and Butt, 2015; Jha et al., 2019).

**Figure 1:**
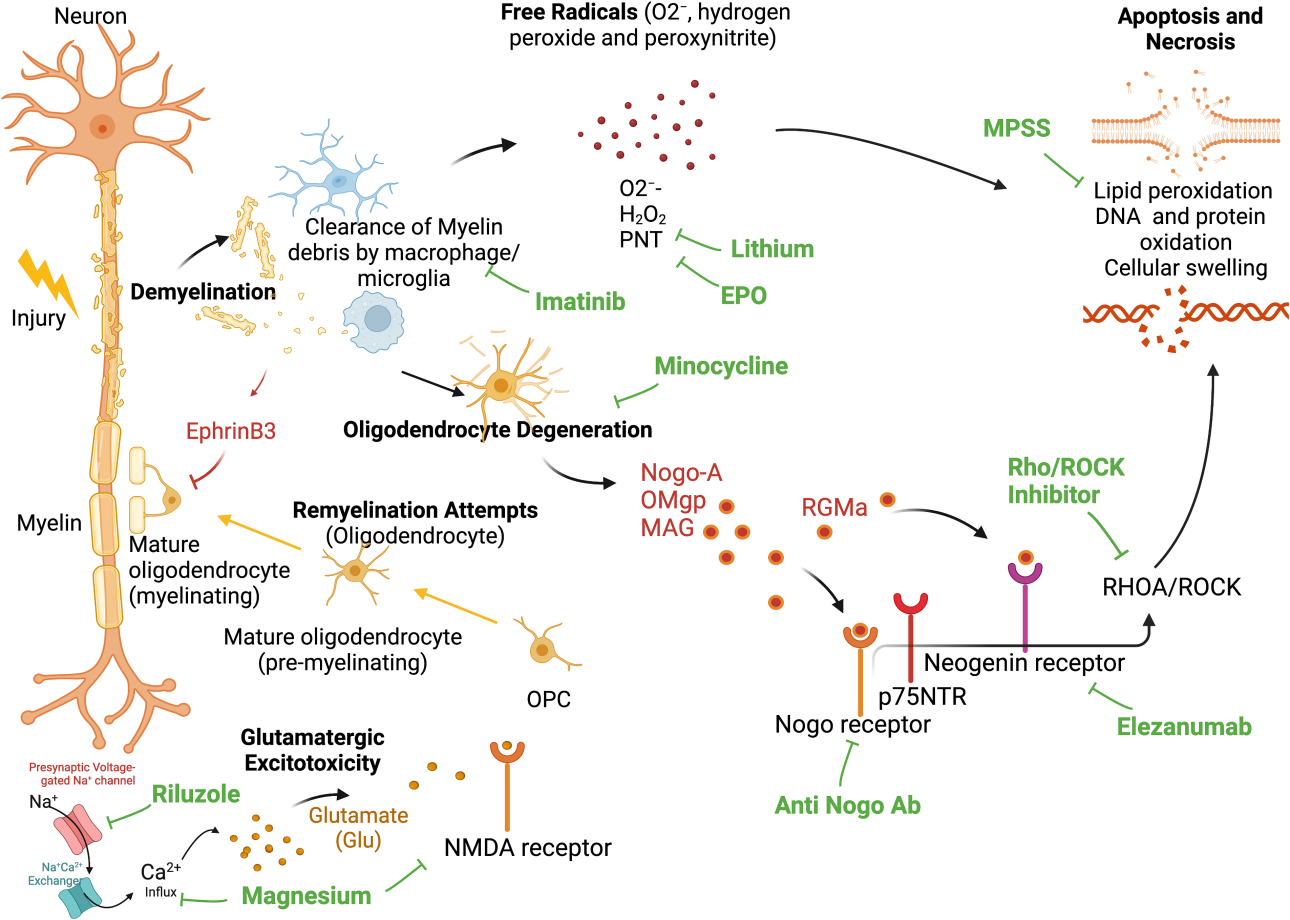
Secondary Injury and site of action for neuroprotective and neuroregenerative therapies. Demyelination leads to invasion of phagocytic inflammatory cells (Macrophage and Microglia), that clear myelin debris and release cytotoxic byproducts, including free radicals (O2−, hydrogen peroxide and peroxynitrite), causing additional necrotic, and apoptotic cell death due to DNA-, protein-, and lipid oxidation. Activated microglia and macrophages additionally propagate the inflammatory response and contribute to ongoing apoptosis of oligodendrocytes. Degenerated oligodendrocytes release neurite outgrowth inhibitor A (Nogo-A), oligodendrocyte-myelin glycoprotein (OMgp), and myelin-associated glycoprotein (MAG). These molecules can all bind the Nogo receptor and p75 neurotrophin receptor (p75NTR) to activate RHOA and Rho-associate protein kinase (ROCK), which further causes neurite retraction and apoptosis. Oligodendrocyte precursor cells (OPC) can differentiate into mature oligodendrocytes and remyelinate these injured axons. However, remyelination is inhibited by the presence of EphrinB3 in myelin debris. This leads to deprived remyelination post-injury. EPO, Erythropoietin; MPSS, Methylprednisolone Sodium Succinate; PNT, peroxinytrite.

The loss of myelin sheath leads to impairment of neural transmission. Phagocytic inflammatory cells can clear myelin debris at the injury site but can also induce further damage to the spinal cord (Figure 1) through the release of cytotoxic by-products, including free radicals (O2^−^, hydrogen peroxide and peroxynitrite), causing additional necrotic and apoptotic cell death due to DNA protein oxidation, as well as lipid peroxidation (Dizdaroglu et al., 2002). Additional molecules present during the myelin and oligodendrocyte degradation process can further hinder axonal regeneration. These molecules include neurite outgrowth inhibitor A (Nogo-A), oligodendrocyte-myelin glycoprotein (OMgp), and myelin-associated glycoprotein (MAG). All of these can bind to the Nogo receptor and p75 neurotrophin receptor (p75NTR; also known as tumor necrosis factor receptor 16), thereby activating RHOA and Rho-associated protein kinase (ROCK). This activation finally leads to neurite retraction and apoptosis (Filbin, 2003).

While the secondary injury advances and a significant proportion of the spinal cord white matter is destroyed, a viable subpial rim of demyelinated axons can persist (Fehlings and Tator, 1995). These neurons remain vulnerable to subsequent injury and may undergo a progressive organized process of axonal death (also known as Wallerian degeneration) (Fehlings and Tator, 1995). Oligodendrocyte precursor cells (OPC) have the capacity to differentiate into mature oligodendrocytes and subsequently remyelinate axons. However, the process of remyelination is contingent upon a well-coordinated inflammatory response involving macrophages, lymphocytes, and astrocytes. The presence of EphrinB3, derived from myelin debris, inhibits this process (Syed et al., 2016). Consequently, this may result in inadequate remyelination following injury, subsequently impairing functional recovery (Jeffery and Blakemore, 1997). Finally, in the subacute to chronic phases of tSCI, reactive astrocytes form a glial scar around the injury site, which can further inhibit axonal regeneration and contribute to the permanence of the injury.

The chronic phase is distinguished by ongoing efforts in remyelination, vascular reorganization, and remodeling of neural circuits (Kwon et al., 2004). Due to a reduction in the inflammatory response, the harsh microenvironment of the injury stabilizes over time. However, the consequences of the secondary injury frequently lead to ongoing neurological deficits (Jeffery and Blakemore, 1997). The extensive cell death and degeneration contribute to the “*ex vacuo* phenomenon” (i.e., loss of tissue volume), resulting in the formation of cystic cavities. These microcystic cavities contain extracellular fluid, macrophages and are encircled by a mesh-like formation of astrocytes (Norenberg et al., 2004; Hejrati and Fehlings, 2021). This astrocytic border plays a major role as an inhibitor to axonal regrowth (Chio et al., 2022). Due to impaired neuronal pathways and inhibiting neurite outgrowth, changes in the spinal cord proximal and distal to the injury site occur, thereby diminishing anatomical plasticity (Ahuja et al., 2017).

**Figure 2:**
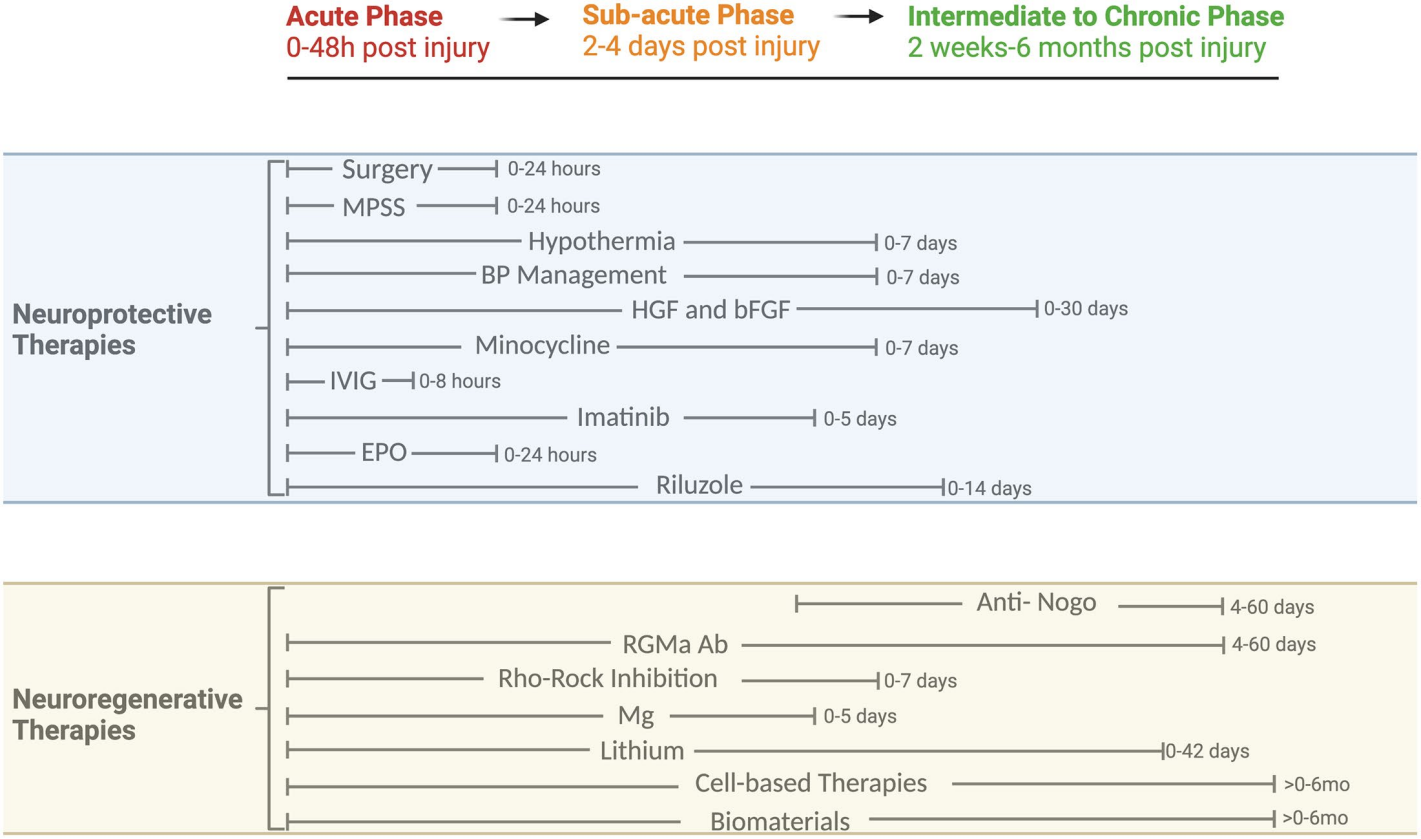
Phases of tSCI and timeline of neuroprotective and neuroregenerative therapies. Overview of clinical and preclinical therapies and their respective therapeutic windows. MPSS, Methylprednisolone Sodium Succinate; BP, Blood Pressure; HGF, Hepatocyte growth factor; bFGF, Basic fibroblast growth factor; IVIG, Intravenous Immunoglobulin G; EPO, Erythropoietin; Nogo, Oligodendrocytes release neurite outgrowth inhibitor A; RGMa, Repulsive Guidance Molecule A; Rho/ROCK, Rho-associated coiled-coil protein kinase; Mg, Magnesium.

As seen above, a myriad of factors have been explored and may impose a relevant contribution to the pathophysiology of tSCI. Particularly during the secondary injury phase, a multitude of potential working points have emerged, which render a single therapeutic solution for the management of tSCI unlikely. Based on the pathophysiology of tSCI, a combination of therapeutic modalities is most likely needed for successful results aimed at attenuating the secondary injury (Figures 1, 2).

## Clinical management

### Timing of surgery

Within the concept of “Time is Spine,” early decompressive surgery stands as a pivotal neuroprotective measure embraced by spinal surgeons (Badhiwala et al., 2021). In essence, surgery seeks to realign the spinal column, restore spinal stability, and alleviate compression (either bony or disco-ligamentous) on the spinal cord. This generally involves open or closed reduction and decompression, coupled with instrumented fusion to stabilize the spinal column in its anatomical position. From a neuropathophysiological standpoint, persistent compression of the spinal cord is believed to worsen local spinal cord ischemia, consequently amplifying the secondary injury (Dimar et al., 1999). Evidence from a systematic review and meta-analysis of preclinical studies supports the notion that prolonged spinal cord compression is typically associated with adverse outcomes, including impaired neurobehavioral recovery and disturbances in blood flow (Batchelor et al., 2013). Accordingly, in the clinical setting, strong evidence supports the role of early decompressive surgery within the first 24 h of tSCI, as this has been associated with enhanced neurological outcome and improved American Spinal Injury Association (ASIA) Impairment Scale (AIS) grade conversion at 6-month follow-up (Fehlings et al., 2012). More recently, a pooled analysis of 1,548 patient data (Badhiwala et al., 2021) showed that early decompression within 24 h of acute SCI is associated with improved sensorimotor recovery and AIS grade recovery at 1 year after surgery. Recent studies have explored the role of “ultra-early” surgery, suggesting potential benefits if surgery is conducted within 8 h of injury (Jug et al., 2015; Grassner et al., 2016). In contrast, the Observational European Multicentre study on the efficacy of acute surgical decompression after traumatic Spinal Cord Injury (SCI-POEM) study (Hosman et al., 2023), suggested that early surgical decompression (≤ 12 h) does not lead to clinically relevant or statistically significant neurological improvements at 12 months post-injury. These results, however, should be interpreted with caution, based on heterogenous patient population, issues related to ceiling effects, and a non-negligible loss to follow-up (Van Middendorp et al., 2013; Hosman et al., 2023).

Ultimately, early decompression surgery still stands as a fundamental cornerstone in the management of SCI, has been recognized globally, and is therefore incorporated as a clinical recommendation in the most recent AO-Spine practice guidelines (Fehlings et al., 2017), with the updated AO Spine/Praxis Spinal Cord Institute Guidelines recently published (Fehlings et al., 2024). Notably, in low and middle-income countries (LMICs), the adoption of early decompressive surgery as a globally standardized practice faces barriers such as resource/logistical constraints and insufficient knowledge transfer, leading to an unlikelihood of early decompression (<24 h) in nearly 60% of institutions in LMICs (Hejrati et al., 2022).

### Surgical considerations

In analogy to traumatic brain injury, where an expansile duroplasty is almost invariably carried out as part of a decompressive craniectomy (Sahuquillo and Dennis, 2019), the role of expansile duroplasty for the surgical management of tSCI is increasingly discussed (Aarabi et al., 2022; Garg et al., 2022; Saadoun et al., 2023).

Under normal conditions, the spinal cord is surrounded by cerebrospinal fluid (CSF), creating a fluid interface between the spinal cord and the dura mater. Under physiologic conditions, intraparenchymal pressure equals the CSF pressure in the spinal subarachnoid space (SAS) (Papadopoulos, 2015; Hogg et al., 2019). Any post-traumatic hemorrhage or cytotoxic/vasogenic edema causing spinal cord volume increase results in proportional CSF displacement (Aarabi et al., 2022). Swelling may lead to the spinal cord occupying the entire cross-sectional area of the thecal sac, thereby resulting in an increase of intraspinal pressures (ISP) and potentially reducing spinal cord perfusion pressures (SCPP) and a compromise of spinal cord autoregulation (Gallagher et al., 2019; Hogg et al., 2019).

While bony decompression remains the established surgical standard in the management of tSCI where there is osseus compression of the spinal cord, recent findings challenge its effectiveness in adequately alleviating ISPs under certain circumstances (Zhu et al., 2019). Achieving sufficient decompression by removing all extrinsic and intrinsic pressure from the spinal cord in conjunction with anatomic realignment and fusion, may normalize ISPs (Zhu et al., 2019). In patients with inadequate decompression, as seen in postoperative magnetic resonance imaging (MRI)‘s by a depleted perimedullary SAS, additional expansion duraplasty may be necessary (Aarabi et al., 2022). Aarabi et al. (2019) investigated the influence of anterior cervical discectomy and fusion (ACDF), anterior cervical corpectomy and fusion (ACCF) with or without laminectomy as well as stand-alone laminectomy on the rates of successful spinal cord decompression in patients with blunt cervical tSCI and AIS grades A-B. The likelihood of achieving a complete decompression, as indicated by a patent SAS in the region of the contused and swollen spinal cord on postoperative MR-imaging, was significantly associated with a laminectomy when added to an ACDF, ACCF or even as a stand-alone procedure, (odds ratio 4.85; 95% CI 2.2–10.6; *p* < 0.001). Nevertheless, in seven out of 63 patients (11%) who had unsuccessful decompression, even laminectomy was inadequate to restore the patency of the SAS. It has been recently shown that the presence/absence of a perimedullary SAS at the level of injury may impact AIS grade conversion, whereby nearly 60% of the patients with adequate decompression (patent SAS) experienced AIS grade conversion, whereas only 18.5% of those with inadequate decompression (impatent SAS) demonstrated such conversion (*p* < 0.001) (Aarabi et al., 2017).

A retrospective, single-center study by Aarabi et al. showed an effacement of the perimedullary SAS in 10% of 104 patients with AIS grades A–C, despite achieving satisfactory bony decompression through multi-level laminectomy (Aarabi et al., 2022). In these cases, an expansile duraplasty may potentially address the issue of spinal cord edema, resulting in a reduction of ISPs, as proposed by real-time ISP-monitoring, revealing that laminectomy without duraplasty fails to normalize ISP (Zhu et al., 2019).

In support of this, studies by Phang et al. (2015) and Zhu et al. (2019) underscore the necessity of laminectomy with duraplasty for meaningful reductions in ISP thereby increasing SCPPs. Interestingly, in 2019, Hogg et al. (2019) investigated the influence of 28 clinical and MRI features in 64 patients with tSCIs on ISPs and SCPPs, 24 h after surgery. Among these factors, only five were significantly correlated with a decrease in ISPs, with expansion duroplasty being notably influential (*p* < 0.05). The ongoing prospective, multicenter, randomized controlled superiority DISCUS (Duroplasty for injured cervical spinal cord with uncontrolled swelling) trial is currently examining the role of expansile duroplasty in patients with AIS grades A-C SCI’s (Saadoun et al., 2023).

### The role of intraoperative ultrasound

Intraoperative ultrasound (IOUS) is an effective tool, which can visualize the extent of surgical decompression in patients with tSCI. A patent perimedullary SAS and a pulsating cord may be indicators for a sufficiently decompressed spinal cord (Hogg et al., 2019; Aarabi et al., 2022). Furthermore, the exact location of the compressing element can be visualized and ultimately addressed. Finally, real-time IOUS can further visualize intradural and/or intramedullary hematoma, thereby further enhancing surgical efficacy (Hogg et al., 2019). More evidence is needed from clinical studies, regarding the net benefit of the use of IOUS to generate favorable patient outcomes.

### Hemodynamic management

It is generally understood that hemodynamic management, along with cardiac and respiratory monitoring within the first few days after SCI may decrease the risk of secondary injury due to spinal cord ischemia (Gee and Kwon, 2022). Moreover, it is though that drugs for the treatment of tSCI better reach their acting site with an increase of MAPs (Saadoun and Papadopoulos, 2020). The 2013 AANS/CNS (American Association of Neurological Surgeons/Congress of Neurological Surgeons) guidelines recommend hemodynamic monitoring and blood pressure management in an ICU, or an analogous monitoring setting (Walters et al., 2013). As such, maintenance of mean arterial pressure (MAP) targets between 85 to 90 mmHg for the first 7 days following tSCI and avoidance of systolic blood pressures (SBPs) <90 mmHg are recommended (Ahuja et al., 2017). Nevertheless, resource limitations, particularly in LMICs, and a paucity of high-quality evidence related to its efficacy, pose challenges to its implementation into clinical practice (Hejrati et al., 2022).

In order to maintain an adequate blood pressure level, both crystalloids or vasopressors such as phenylephrine or norepinephrine are used. As hemodynamic monitoring requires invasive monitoring and potential intravenous administration of fluids and vasopressors, it may pose a challenge for early mobilization. This in turn may potentially increase the risk for complications associated with immobilization (Saadoun and Papadopoulos, 2020; Gee and Kwon, 2022).

While the great majority of hemodynamic studies in the field of tSCI clinical research have been using MAPs as a variable to manage blood pressure (Papadopoulos, 2015; Hogg et al., 2019), recent data has demonstrated that adhering to SCPPs may be more predictive for neurological recovery than MAPs (Gee and Kwon, 2022). SCPP is defined as the difference between MAP and the pressure within the intrathecal space. While the concept of SCPP-targeted hemodynamic management considers the intraspinal pressure, it requires intrathecal pressure monitoring (Saadoun and Papadopoulos, 2020). Two approaches have been employed to assess pressure within the intrathecal space. One involves the direct placement of a probe at the lesion site during decompressive surgery, while the other uses a pressure probe inserted at the lumbar intrathecal space, the latter offering the opportunity for CSF diversion, in case needed. Of note, the lumbar intrathecal probe measures the pressure below the level of injury, thereby potentially underestimating the actual pressure present at the injury site due to swelling of the spinal cord and its expansion against the dura mater (Gee and Kwon, 2022). Ultimately SCPPs can either be optimized by adjusting MAPs or by draining CSF with the intention of decreasing the pressure in the intrathecal space. The combination of the two methods offers the opportunity to enhance spinal cord perfusion (Gee and Kwon, 2022). While ISP monitoring at the site of injury allows for direct interpretation of the pressure environment of the spinal cord, it has been associated with higher rates of pseudomeningoceles and CSF leakage, leading to a higher risk of meningitis (Gee and Kwon, 2022). Therefore, measuring intrathecal pressures in the lumbar cistern is considered safer.

Squair et al. (2017) investigated SCPP-targeted hemodynamic management in 92 tSCI patients over a one-week period post-injury. The SCPP in individuals exhibiting gradual improvement in neurological function experienced fewer instances of pressure drops below 50 mmHg, compared to individuals without improvement (*p* = 0.012).

Ultimately, while there is limited evidence on the utility of SCPP-guided hemodynamic management, it may offer the opportunity to personalize blood pressure management and CSF drainage in individuals suffering from tSCI by allowing clinicians to account for the variable intrathecal pressure environment (Iovine et al., 2022).

### Hypothermia

Hypothermia is known to reduce metabolic demands of central nervous system (CNS) tissues and decrease inflammatory cell activation, thereby protecting from neuronal cell death in traumatic brain injury (TBI) (Ahuja et al., 2017). Preclinical studies have been promising, showing improved neurological outcomes and few side effects after TBI (Ransom et al., 2022). Four clinical studies have been conducted to date, applying either local (epidural, subarachnoid) or systemic hypothermia (surface cooling) (Ransom et al., 2022). Systemic hypothermia can be induced either via cooling blankets, or endovascular heat exchange catheters. Local hypothermia is induced via an epidural heat exchanger or subarachnoid cold solution perfusion (Ransom et al., 2022). 43% of patients (*N* = 35) with tSCI treated with systemic hypothermia had an improvement in the AIS grading, whereas 58% (*n* = 14) of patients who were treated with local hypothermia experienced an improvement in AIS grading (Ransom et al., 2022). Complications, which have been reported to occur due to hypothermia, include pneumonia (60% in the systemic group versus (vs.) 28% in the local group) and atelectasis (83% in the systemic group vs. 36% in the local group). Other reported complications were thromboembolic complications (24% in the systemic group) and wound infections (5% in the local group). A negative impact on neurological function has not been observed.

Randomized controlled trials investigating on the safety and effectiveness of hypothermia for acute tSCI are required. A prospective multi-center trial from the University of Miami, investigating the safety profile and efficacy of modest (33°C) intravascular hypothermia following cervical tSCIs is in progress (NCT02991690). First results are expected in September 2024.

## Neuroprotective therapies

### Steroids

In an effort to reduce the inflammatory response during the secondary injury phase of tSCI (Ducker and Hamit, 1969), the impact of Methylprednisolone Sodium Succinate (MPSS), a potent synthetic glucocorticoid, believed to enhance neuronal anti-inflammatory cytokines and inhibit lipid peroxidation of cell membranes as well as reducing edema, has been explored in numerous RCTs (Bracken et al., 1990, 1992, 1998; Pointillart et al., 2000). Based on the results from the NASCIS II and III trials, MPSS has been recommended for SCI management due to its demonstrated effectiveness in short-term motor improvement (Bracken et al., 1992, 1998). However, a 2016 meta-analysis (Evaniew et al., 2016) indicated that while it provides short-term benefits, there is no improvement in long-term motor outcomes, and its use has been associated with an increased risk of gastrointestinal bleeding. A 2019 meta-analysis (Liu et al., 2019) found no motor or ASIA score improvement at short- or long-term follow-up. In a more recent systematic review and meta-analysis (Sultan et al., 2020), the utility of steroids within the first 8 h following SCI failed to show a statistically significant short- or long-term improvement in patients’ overall motor scores or neurological outcomes compared to controls who were not administered steroids. In the same comparison, an increased risk of pneumonia and hyperglycemia was observed in contrast to the control group. A systematic literature review by Fehlings et al. was undertaken in 2017 to formulate AO Spine practice guidelines for the utilization of MPSS after tSCI. Following the GRADE approach (Grading of Recommendation, Assessment, Development and Evaluation) the authors propose a 24-h infusion of high-dose MPSS within 8 h after onset of SCI as a treatment option (Fehlings et al., 2017). This suggestion was justified by the pooled analysis, which showed no statistically significant differences in harmful side effects while showing modest improvements in ASIA motor scores if MPSS is given according to the NASCIS II protocol within 8 h following injury (Fehlings et al., 2017).

A new, case-matched dataset (Geisler et al., 2023) pooled from the NASCIS II (Bracken et al., 1990) and the Sygen (Geisler et al., 2001) databases was used to investigate the effect of MPSS on SCI with contemporary clinical scales and exclusion criteria. The new pooled dataset from the NASCIS II contained 31.6% fewer patients than the 1990 dataset, excluding patients with injury levels caudal to T10, lower-extremity ASIA motor scores ≥46, Glasgow Coma Scale scores ≤11, and age < 15 or > 75 years. It revealed that the administration of MPSS following tSCI did not demonstrate any improvement in neurological recovery at 26 weeks. This analysis provides evidence that the positive *post hoc* sub-subgroup results initially reported in 1990 by NASCIS II resulted from randomization imbalance in the injury severity groups and inclusion of patients (e.g., injury levels caudal to T10, lower ASIA motor scores ≥46), which nowadays would not be diagnosed with a tSCI.

The latest meta-analysis (Guan et al., 2023), scrutinized eight guidelines, formulated between 2008 and 2020, specifically addressing the use of MPSS. Among these guidelines, three recommended its use (37.5%, comprising one evidence-based and two consensus-based recommendations), three advised against its use (37.5%, all evidence-based), and two suggested a neutral stance (25%). This gives rise to a discernible incongruence in recommendations regarding steroid administration, with evidence-based guidelines leaning toward avoidance and consensus-based guidelines leaning toward endorsement (Cabrera-Aldana et al., 2017; Li et al., 2018). In light of divergent research findings, the administration of high-dose steroids during the acute phase of tSCI experienced a shift in practice across North American Clinical Trials Network (NACTN) centers, moving away from the use of steroids, particularly between 2009 and 2010. In 2009, 71% of patients received steroids, but in 2010, this trend reversed, with 80% of patients not receiving steroids (Fehlings et al., 2023). This pattern persisted until today, turning it into a rarely used therapeutic option (Fehlings et al., 2023; Hejrati et al., 2023).

### Hepatocyte growth factor

Hepatocyte Growth Factor (HGF), recognized as a mediator of inflammatory responses to tissue injury, has emerged as a robust neurotrophic factor within the CNS (Kitamura et al., 2011). Specifically, the application of exogenous HGF via a herpes simplex virus 1 vector into the injured spinal cord of adult rats following SCI has demonstrated notable neuroprotective and antiapoptotic effects, by amplification of angiogenesis around the lesion center (Kitamura et al., 2007). Following a randomized double-blind placebo-controlled phase I/II study (Nagoshi et al., 2020), favorable motor functional recovery was observed in patients, who underwent intrathecal KP-100 (recombinant HGF) administration during the acute stage of tSCI. The KP-100 group exhibited superior motor scores in the lower extremities compared to the placebo group, and a favorable safety profile with no harmful adverse events recorded during the observational period. Considering both safety and efficacy, KP-100 could be a beneficial drug for SCI, but ultimately a larger phase III trial would be required to evaluate its efficacy.

### Fibroblast growth factor

Basic fibroblast growth factor (bFGF; also known as fibroblast growth factor 2) has shown promise in the context of tSCI. It plays a role in promoting cell proliferation, differentiation, and survival, which are crucial for neural regeneration (Santa-Otalla and Covarrubias, 1999). Research in animal models suggests that bFGF can contribute to neuroprotection against excitotoxicity and can reduce injury-mediated free radicals (Teng et al., 1999; Rabchevsky et al., 2000). Transient infusion of bFGF can promote axon regeneration and functional recovery in rats (Liu et al., 2011). However, bFGF has limited capacity to penetrate the intact BSCB due to its macromolecular dimensions. This limitation restrains its utility as a viable therapeutic agent for neurological applications (Song et al., 2002). Accordingly, a small synthetic molecule, termed SUN13837, characterized by its properties to mimic the beneficial effects of bFGF, has been proposed as a neuroprotective therapeutic, in particular due to its small lipid-soluble molecules, which render it more likely to penetrate the BSCB (Imagama et al., 2020). A recent randomized placebo-controlled trial (ASCENT-ASCI) was conducted to assess the efficacy of SUN13837 in individuals with cervical tSCI and AIS grade A lesions. The principal finding of this study suggests that SUN13837 may enhance neurologic recovery. However, it is important to note that these improvements did not translate into significant clinical benefits (Levinson, 2018). In 2015, an open-label randomized rehabilitation-controlled study was initiated involving surgical implantation of SC0806, a biodegradable implant incorporating heparin-activated Fibroblast Growth Factor 1 (FGF1) and nerve implants (NCT02490501). The primary safety and efficacy endpoints include the rates of adverse events and improvements in motor evoked potentials, respectively. Results are still pending.

### Minocycline

Minocycline, a structural analogue of the antibiotic tetracycline, serves a role in the context of tSCI through diverse mechanisms. These include effects by inhibition of both intrinsic and extrinsic pathways of apoptosis, as well as exerting anti-inflammatory effects through modulation of microglia, cytokines and lipid mediators (Chen et al., 2000). Minocycline further protects neurons from N-methyl-D-aspartate (NMDA)-mediated glutamate excitotoxicity of the injured spinal cord tissue (Tikka and Koistinaho, 2001). In rodent models of acute tSCI, it has demonstrated neuroprotective effects by reducing oligodendrocytic apoptosis and attenuation of local inflammation (Lee et al., 2003; Wells et al., 2003). Moreover, it was observed that administration of minocycline reduces the volume of the spinal cord lesion area and prevented axonal death within the rubrospinal tract in mice with acute tSCI (Wells et al., 2003).

In a phase II placebo-controlled randomized study (*n* = 52, ASIA A-D) (Casha et al., 2012), the administration of minocycline for 7 days after tSCI resulted in significant improvement of 6 points in ASIA motor scores at one-year follow-up when compared to the placebo group (95% CI –3 to 14, *p* = 0.20). Patients were administered 200 mg twice-daily intravenously, maintaining steady-state concentrations of 12.7 μg/mL in serum and 2.3 μg/mL in cerebrospinal fluid, matching effective serum levels observed in animal studies. Overall, the treatment showed a high safety profile with only one reported adverse event (increased levels of hepatic enzymes). A larger multicenter efficacy trial with patients receiving either placebo or intravenous minocycline after tSCI is currently ongoing (NCT01828203).

### Immunoglobulin G

Intravenous Immunoglobulin G (IVIG) has attracted interest as an immunomodulatory therapy, after initial enthusiasm for MPSS has decreased due to associated complications and insufficient proof of efficacy for the latter. IVIG has been accepted as an FDA-approved immunomodulatory therapy employed in the management of autoimmune disorders (such as Guillain-Barré syndrome). It is thought to mediate its immunomodulatory effects by neutralizing and enhancing clearance of auto-antibodies, interfere with the complement system, modulate B- and T-cell function, inhibit leukocyte migration, induce leukocyte apoptosis and potentially suppress cytokine levels, inflammatory processes, which to some extent are thought to be involved during the secondary injury response following tSCI (Dizdaroglu et al., 2002; Tzekou and Fehlings, 2014).

In a rat model of tSCI using a reproducible cervical clip compression-contusion model, a single bolus of 2 g/kg of human IgG was administered at intervals of 15 min, 1 h, or 4 h following injury (Chio et al., 2021). Notably, animals in the treatment arm demonstrated improved spinal cord tissue preservation as well as neurobehavioral recovery at 8-weeks following injury (Chio et al., 2021). Other neuroprotective effects associated with IVIG therapy following acute tSCI are thought to be mediated through improved myelin preservation, although the mechanism behind this neuroprotective effect in tSCI is still unknown (Nimmerjahn and Ravetch, 2008; Gillespie and Ruitenberg, 2022). Encouraged by these promising preclinical findings, a phase I/IIa clinical trial (ACTRN12616001385437) is currently enrolling patients aimed at scrutinizing the pharmacokinetic attributes and therapeutic efficacy of IVIG in the context of tSCI.

### Imatinib

Imatinib (marketed as Gleevec^®^, Novartis) acts as a tyrosine kinase inhibitor with an established clinical use for the treatment of Bcr/Abl-expressing leukemias and c-Kit-expressing gastrointestinal stromal tumors (Abrams et al., 2012). In animal models of tSCI, treatment with imatinib has been demonstrated to reduce intramedullary hemorrhage, edema as well as inflammation, microstructural processes capable of adversely affecting functional recovery following tSCI (Popovich et al., 1993; Flanders et al., 1996). In a rat-model of tSCI, oral imatinib treatment for a duration of 5 days, initiated 30 min post-injury, showed significant improvements of the BSCB integrity by inhibition of inflammatory c-Kit and c-Fms triggered cytokine production in mast cells and macrophages, which was associated with improvements in hindlimb locomotor function (Abrams et al., 2012). A subsequent study using a mouse model of tSCI demonstrated the effectiveness of imatinib in reducing inflammation (Liu et al., 2020), inducing apoptosis in cells contributing to the fibrotic scar (Yao et al., 2022), diminishing neuronal apoptosis resulting from oxidative stress (Liu et al., 2020) and improving locomotor recovery (Yao et al., 2022).These findings highlight imatinib as a potential target for the treatment of tSCI in humans. An ongoing Phase II non-randomized clinical trial (NCT02363361) is assessing the safety, tolerability, and blood levels of oral imatinib treatment in patients with cervical tSCI. The results are pending publication.

### Erythropoietin

Erythropoietin (EPO), is a glycoprotein hormone and hematopoietic cytokine which was originally identified as a kidney- derived stimulator of erythroid progenitor cell proliferation and differentiation (Ratcliffe, 1993). It is also involved in heme biosynthesis and the production of hemoglobin, as well as osteogenic and endothelial differentiation of multipotent mesenchymal stem cells leading to bone remodeling and angiogenesis (Sergio and Rolando, 2022). Despite its broad spectrum of characteristics, concerns exist regarding the risk for thrombosis and adverse events with EPO administration in normonemic adults (Hemani et al., 2021).

Animal tSCI studies where EPO was administered (Boran et al., 2005; Cerri et al., 2012) demonstrated motor improvements of hindlimb motor function as measured with the locomotor BBB (Basso, Beattie, Bresnahan) scale. The neuroprotective properties of EPO administration were further documented in a transient global spinal ischemia rabbit model (by occlusion of the abdominal aorta for 20 min), with animals receiving human recombinant erythropoietin (hR-EPO) exhibiting significantly improved neurological scores according to the criteria of Drummond and Moore (Drummond and Moore, 1989) and reduced motoneuron apoptosis when compared to the control group (Celik et al., 2002). Although the therapeutic benefits of EPO have been demonstrated, the mechanisms underlying these results remain to be established. More recent animal studies suggest that EPO may further counteract inflammation (Shan et al., 2023), as well as mitigate oxidative stress following tSCI (Yazihan et al., 2008; Jin et al., 2014).

In a pilot RCT involving hr-EPO, 20 patients with cervical tSCI were randomly assigned to either receive hR-EPO at 500 IU/mL with methylprednisolone or methylprednisolone alone, within 8 h following injury (Alibai et al., 2015). However, this study did not show significant improvements in functional outcomes in the treatment arm. The most recent RCT involving 54 patients with tSCI, investigating the combined effects of EPO and methylprednisolone (Ganjeifar et al., 2021), replicated earlier findings demonstrating an absence of significant differences in functional outcomes as indicated by the AIS grade and 1-year mortality (OR = 0.83, 95% CI = 0.25-2.74, *p* = 0.76).

### Riluzole

Riluzole is a sodium channel blocker that is FDA-approved for the use in amyotrophic lateral sclerosis (ALS). Preclinical evidence strongly supports the neuroprotective effects of riluzole in the context of SCI, as it inhibits continuous activation of neuronal voltage-gated sodium channels, preventing cellular swelling and death, alongside diminishing excitotoxicity (Wilson and Fehlings, 2014). *In vitro* experiments by the Fehlings group (Wu et al., 2014) revealed that a reduction in extracellular sodium concentrations improved neurophysiologic function in injured dorsal spinal cord columns from adult rats, providing a basis for investigating sodium channel blockage as a mean to mitigate secondary injury after tSCI.

Expanding upon these observations, a subsequent study investigated the impact of riluzole, phenytoin, and CNS5546A (a sodium channel blocker with NMDA antagonistic activity) in a rat model of cervical SCI (Schwartz and Fehlings, 2001). When administered 15 min post-SCI, only riluzole exhibited a notable improvement in functional neurological recovery, as evidenced by enhanced hindlimb function, strength and coordination. Histological examination further showed larger volume of preserved CNS tissue, increased numbers and size of neurons, and reduced cavity area in animals subjected to riluzole treatment. Numerous studies across different animal species have demonstrated riluzole’s neuroprotective and functional recovery-promoting effects in various spinal cord injury models (Stutzmann et al., 1996; Lips et al., 2000; Mu et al., 2000).

These findings provide a compelling rationale for investigating riluzole in clinical trials, such as the phase I prospective multicenter study by Grossman et al. (2014), that showed an improvement in ASIA motor scores in patients with cervical SCIs, 90 days post-riluzole treatment, compared with non-treated counterparts matched from a historical registry cohort. The most recent study named Riluzole in Spinal Cord Injury Study (RISCIS) (Fehlings et al., 2023), compared individuals with SCI and AIS grades A-C, who were randomly assigned within <12 h of injury to receive either riluzole for 14 days, or a placebo. While the primary efficacy endpoint, indicated by the change in Upper Extremity Motor scores at 180 days was not met, the study yielded promising outcomes. Specifically, AIS A patients treated with riluzole exhibited a higher average gain in neurological levels after 6 months when compared to those, who received a placebo (mean 0.50 levels gained vs. 0.12 in placebo; d: 0.38, CI: -0.2-0.9) and all AIS grades A-C treated with riluzole showed significant gains in functional recovery depicted by Spinal Cord Independence Measure (SCIM), Lower Extremity Motor (LEM) scores and Short Form 36 Version 2 (SF-36v2™) (Fehlings et al., 2023). A phase 2 double-blind randomized controlled dose-escalation and efficacy trial (identifier NCT02859792) is currently underway, looking at the effects of riluzole on spasticity for the treatment of tSCI.

## Neuroregenerative therapies

### Anti-Nogo antibodies

Nogo-A is a membrane-bound protein, which can be found on the surface of oligodendrocytes. It acts as a strong inhibitor of neurite growth thereby hindering neuroregeneration (Kucher et al., 2018). After promising results in rodents and non-human primates were demonstrated in 2018, Kucher et al. (2018) examined the safety and efficacy of anti-Nogo-A antibody treatment in humans suffering from acute and subacute AIS grade A SCI. Anti-Nogo-A was injected intrathecally in order to bypass the BSCB. The study group showed that Anti-Nogo-A antibodies are a safe treatment option, however, as far as the follow-up of one year showed, paraplegic patients did not recover significantly more motor function. Tetraplegic patients showed some increase in mean motor scores, with 3 out 19 patients gaining more than 10 points in ASIA motor scores at week 48. The favorable results from this phase I trial have set the stage for a multicenter phase II RCT, which has recently completed enrolment of patients with cervical SCIs (NCT03935321). The results are anticipated to be published in 2024.

### Repulsive guidance molecule A

Following tSCI or TBI, RGMa is upregulated in lesioned areas of rodents, non-human primates, and the human spinal cord where it is thought to act as an inhibitor to neurite growth by binding to its neuronal receptor Neogenin (Mothe et al., 2017; Jacobson et al., 2021). In non-human primates with thoracic hemicompression, Jacobson et al. (2021) could demonstrate that Elezanumab (a human anti-RGMa monoclonal antibody) improves motor function when administered within 24 h post injury and that it promotes neuroplasticity, increasing its potential to positively impact recovery post SCI. Recently, Mothe et al. (2022) found that delayed systemic administration of Elezanumab (a human anti-RGMa monoclonal antibody) promotes neural tissue repair, regeneration, and recovery in a clinically relevant spinal cord contusion/compression rat model of cervical SCI. Elezanumab increased corticospinal and serotonergic axonal plasticity and the formation of synaptic connections caudal to the cervical lesion. An ongoing double-blind phase II RCT (NCT04295538) of Elezanumab in patients with tSCI and AIS grades A or B will assess, whether inhibition of RGMa provides a therapeutic benefit for patients with tSCIs.

### Rho-ROCK inhibitor

Rho, an intracellular GTPase and key mediator in the neuronal apoptotic cascadehas been shown to impede axonal regrowth following tSCI (Sung et al., 2003). Rho-associated coiled-coil protein kinase (ROCK) is a downstream effector of Rho (Kimura and Horikoshi, 2023) with excessive Rho/ROCK activity having implications in a variety of neurodegenerative diseases such as epilepsy, Parkinson’s disease, Alzheimer’s disease. As such, the Rho-ROCK cascade has emerged as a potential therapeutic target (Loirand, 2015).

C3 transferase, an enzyme derived from *Clostridium botulinum* bacteria, inhibits Rho activity, thereby enhancing neuronal regeneration and improving behavioral outcomes in animal models of tSCI (Lord-Fontaine et al., 2008; Forgione and Fehlings, 2014). Administration of exogenous C3 to the site of injury has proven to be challenging. This has led to the development of Ba-210, a C3 fusion protein with improved transport characteristics, which can be administered topically onto the dura using a fibrin sealant (Lord-Fontaine et al., 2008).

Expanding on these findings, a phase I/IIa clinical trial was conducted involving patients with AIS A tSCI’s. Trial results confirmed the safe application of an epidural recombinant version of C3 transferase, named VX-210 (Cethrin). The International Campaign for Cures of SCI Paralysis (ICCP) Clinical Guidelines Panel notes that patients with cervical AIS A injuries experience spontaneous improvement of approximately 10 motor points within the first year after tSCI (Fehlings et al., 2011). However, in this study, an average improvement of 21.3 and 27.3 motor points in the 1-mg and 3-mg Cethrin Dose groups, respectively, at 12 months after tSCI were noted. While the findings of this safety trial suggest encouraging motor improvements, the absence of a placebo group limit conclusions related to its effectiveness (Fehlings et al., 2011). Unfortunately, a phase IIb/III randomized, double-blind, placebo-controlled clinical trial (NCT02669849) investigating the efficacy and safety of VX-210, which was administered topically onto the dura at the site of injury during decompressive surgery, was terminated prematurely (Fehlings et al., 2021). An interim analysis did not reveal statistically significant different changes from baseline upper-extremity motor scores at 6 months between the treatment arm and the placebo group (Fehlings et al., 2021). At present and to our knowledge, there is no other study investigating on the role of Cethrin in the setting of tSCI.

### Magnesium

Within the CNS, while not comprehensively understood, magnesium (Mg) plays a pivotal role in preserving calcium (Ca) homeostasis. Consequently, it is involved in neurotransmitter release, the conduction of action potentials, and transmembrane electrolyte influx (Noronha and Matuschak, 2002). Deficiencies may cause an intracellular calcium overload, disturbances in its subcellular distribution and ultimately serotonin and acetylcholine excitotoxicity as well as a decrease in inhibitory amino acids such as y-amino butyric acid (GABA)(Noronha and Matuschak, 2002). Both in animal models of SCI and in humans, decreasing levels of Mg post injury could be observed and were linked to an exacerbation of the secondary injury (Cernak et al., 2000).

Magnesium can function as an NMDA receptor antagonist thereby reducing excitotoxicity. Moreover, it has been suggested to act as an anti-inflammatory agent. Stable CSF levels can be generated by delivering magnesium with an excipient such as polyethylene glycol (PEG) (Ditor et al., 2007). In animal models, the combination of magnesium and PEG (Mg-PEG) has demonstrated the ability to improve tissue sparing and promote behavioral recovery (Kaptanoglu et al., 2003).

In a recent Phase III double-blind trial (Temkin et al., 2007) involving a cohort of 499 patients receiving MgSO4 to maintain a serum magnesium concentration of 1.0-1.85 mmol/L or 1.25-2.5 mmol/L for 5 days following TBI, adverse outcomes (such as seizures and hypotension) and a lack of neuroprotective effects were observed in both the lower and higher dosage treatment groups when compared to the placebo-group. Notably, the intervention group showed a higher mortality rate (HR 2·22 in the higher and 1·33 in the lower dose groups; 95% CI 1.00-5.50 and 0.87-2.10) than the placebo group. In contrast, Dhandapani et al. reported favorable clinical outcomes, regarding Glasgow Coma Scale at 3 months and mortality in 73.3% of patients with TBI after administering MgSO4 within 24 h (pritchard regimen) (Dhandapani et al., 2008). Thus, ideal dosage and timing of administration seem to be crucial to achieve positive effects.

Nevertheless, at present, there is no evidence that additional Mg administration in the early phase after SCI has a positive effect on patient ouctomes. In a more recent study Sperl et al. found an inverse correlation between Mg level at 1 week after SCI and the potential of neurological remission, with significantly lower Mg levels in patients with AIS conversion >1 (Sperl et al., 2019).

In conclusion, the existing data regarding the safety and efficacy of Mg is ambiguous, necessitating further clinical investigations to ascertain whether supplemental Mg administration can indeed enhance neurological outcomes following SCI.

### Lithium

Despite the proposed associations of lithium to mitigate oxidative stress and apoptosis, the specific mechanism by which lithium confers protection against the harmful microstructural effects following tSCI remains poorly understood (Zhao et al., 2022). One potential mechanism of action has been found in the inhibition of pathologically increased levels of Glycogen synthase kinase 3-(GSK-3) following tSCI, which can ultimately induce neurogenesis (Rodriguez-Jimenez et al., 2021).

In a rat contusion model of thoracic tSCI, the combined treatment of lithium and transplanted human neural stem cells (NSCs) provided more substantial improvements in locomotor recovery compared to each treatment alone and the control group (Mohammadshirazi et al., 2019). This observation may be explained by enhanced proliferation and differentiation of transplanted NSCs and greater host motor neuron survival following transplantation (Zhang et al., 2018). In a more recent preclinical study using a rat model of tSCI (Zhao et al., 2022) the effects of intraperitoneal administration of lithium chloride (LiCl) showed that LiCl administration inhibits the expression of pro-inflammatory cytokines, including TNF α, interleukin-6 (IL-6), and interleukin-1β (IL-1 β), thereby showing neuroprotective properties.

A phase I clinical trial (Wong et al., 2011), where 20 patients with a minimum 12-month history of tSCI (AIS A-C) were administered oral LiCl, maintaining serum lithium levels between 0.6-1.2 mmol for six weeks, objectified no severe side effects. Subsequently, a phase II double-blind placebo controlled RCT (Yang et al., 2012) involving 40 patients, with at least a 12-month history of cervical or thoracic tSCI (AIS A-C) was conducted. While lithium did not result in functional improvements, it exhibited a reduction in Visual Analog Scale (VAS) pain scores compared to the placebo arm, suggesting a potential therapeutic utility for the management of neuropathic pain (Yang et al., 2012).

### Rehabilitation

The goal of rehabilitation after SCI lies in preserving and restoring function and preventing secondary complications, as well as enhancing neuroregeneration and task-specific functional recovery by stimulating neural circuits (García-Alías and Fawcett, 2012). With loss of independence, it also supports the reintegration of the patient into society and addresses other practical challenges, such as vocational and financial concerns (Fehlings et al., 2017). Essential elements of rehabilitation include strength training, cardiovascular exercises, respiratory conditioning, transfer or mobility training, and stretching to deter muscle contractures. Notably, physical rehabilitation can induce alterations in cellular signaling and the expression of growth factors (Hwang et al., 2014). Early mobilization has been shown to elevate levels of endogenous growth factors, such as insulin-like growth factor 1, and promote axon regeneration in animal models (Hwang et al., 2014). However, in clinical scenarios, challenges like ventilator dependence, neuropathic and somatic pain, psychosocial issues, and resource constraints can pose difficulties for early mobilization. Despite the tendency to underestimate these substantial clinical barriers, the impact of rehabilitation on neuroregeneration after tSCI has been shown by an increase in locomotor function in clinical studies (Dobkin et al., 2006; Sandrow-Feinberg and Houlé, 2015). A contemporary rehabilitation approach, such as the weight-supported locomotor training (WSLT), utilizes assisted devices like Hocoma’s Lokomat and HealthSouth’s AutoAmbulator, along with therapists, to dynamically support the patient’s weight during locomotion training (Dobkin et al., 2006). The therapy aims to enhance the residual connectivity between regions above the injury and the locomotor central pattern generator (a region of neurons capable of initiating locomotion independently of input from other brain-regions) (Pernía-Andrade et al., 2021). WSLT has demonstrated effectiveness in improving assisted mobility, cardiorespiratory status, and preventing pressure sores and joint-related complications of SCI. In a randomized, single-blinded trial involving 146 participants, comparing 12 weeks of WSLT to a similar intensity of physical rehabilitation, no significant difference in outcomes was found (Dobkin et al., 2006). However, both groups exhibited improvements in locomotion at 6 months, underscoring the importance of intensive rehabilitation (Dobkin et al., 2006). It is important to highlight that multimodal rehabilitation has the potential to promote functional recovery, even in individuals with chronic motor-complete tSCI (Gant et al., 2018).

It is proposed that the CNS undergoes reorganization throughout the processes of acquiring, retaining, and consolidation of motor skills (Tashiro et al., 2021). This overarching idea is condensed in the terms neurorehabilitation and neuroplasticity (Zheng et al., 2020). The considerable overlap in the primary mechanisms driving the effects of both stem cell therapies and neurorehabilitation suggests a synergistic interaction between these two treatment elements, as evidenced by preclinical studies elucidating molecular mechanisms and outcomes (Rando and Ambrosio, 2018). Investigations into combined treatments have highlighted synergistic effects (Tashiro et al., 2016, 2021). While stem cell therapy alone appears to yield limited recovery, the combined impact of regenerative rehabilitation could be crucial in achieving substantial motor and functional recovery after tSCI.

### Cell-based therapies

Following tSCI, nerve conduction is impaired, and regeneration of the neural circuits is limited due to the inhibitory SCI microenvironment. Transplantation of progenitor cells into the lesion, expecting they may undergo differentiation and serve as surrogate neurons, has been explored since the 1990s (Ahuja et al., 2017; Kawai et al., 2021). Transplantation of various cell types has been investigated in animal models, (Table 1). Cell types that have been assessed in preclinical studies include cells like Schwann cells, peripheral nerve grafts, genetically modified fibroblasts, a type of glial cells named olfactory ensheating cells (OECs) and stem cells. Stem Cell Therapy (SCT) indicates a developing treatment paradigm using the differentiation, self-renewal and paracrine capabilities of stem cells to regenerate the injured spinal cord. To date, multipotent stem cells including mesenchymal stem cells (MSCs), neural stem cells (NSCs) and hematopoietic stem cells (HSCs) represent the most investigated cell types, (Table 1). Several trials have examined the safety and initial effectiveness of cell transplantation in individuals with SCI.

**Table 1:** Cell types investigated for SCI treatment.

Type of stem cells	Source	Proposed mechanism of action	Limitations	Application in SCI	References
Induced pluripotent stem cells	Human adult somatic cells	Immunomodulation, axon regeneration and sprouting; lack of immune suppression (with autologous cell sources)	Risk of immune rejection, instability of iPSCs’ genome, potential tumorigenicity	Preclinical	Takahashi et al. (2007), Fu (2013), Ji et al. (2016)
Embryonal stem cells	Human umbilical cord, amnion, chorion, generated from adult somatic cells, Fibroblasts	Potential to generate various cell lines, such as neurons or oligodendrocytes; Axon regeneration and sprouting, myelinogenesis	Potential for immune rejection, ethical considerations related to human embryo usage, risk of tumorigenicity.	Phase I-II	Shroff and Gupta (2015), Bacakova et al. (2018), Hoang et al. (2022), Mousaei Ghasroldasht et al. (2022)
Mesenchymal stem cells	Human bone marrow, umbilical cord blood, myeloid, and adipose tissue	Immunomodulation, axon regeneration and sprouting; capability to generate adipocytes, bone, chondrocytes, rapid proliferation, low immunogenicity	Time-consuming production via liposuction or bone marrow aspirate followed by cultivation	Phase I-II	Vaquero et al. (2018), Cofano et al. (2019), Kazim et al. (2021), Saini et al. (2022)
Neural stem cells	Human spinal cord’s central canal, ventricular system, hippocampus, somatic cell differentiation, and iPSCs.	Immunomodulation, axon regeneration and sprouting; capability to differentiate into neurons, oligodentrocytes and astrocytes	Risk of immune rejection, limited sources	Phase I-II	Martin-Lopez et al. (2021), Hou et al. (2022)
Hematopoietic stem cells	Human placenta, cord blood, adult bone marrow	Capability to differentiate into all cell types of the hematopoietic system	Risk of immune rejection	Phase I-II	Dulak et al. (2015), Oliveira et al. (2021), Mousaei Ghasroldasht et al. (2022), Nikoonezhad et al. (2022)
Olfactory ensheating cells	Olfactory bulb/mucosa	Axon regeneration, growth, and sprouting	Risk of co-transplantation of respiratory epithelium resulting in intramedullary cyst formation, immune rejection	Phase I-II	Mackay-Sim et al. (2008), Dlouhy et al. (2014)
Schwann cells	Sciatic nerve	Myelinate axons, axon regeneration and sprouting, modification of glial scar	Risk of immune rejection	Phase I-II	Oudega and Xu (2006), Ching et al. (2018), Pearse et al. (2018), Levi et al. (2019), Gant et al. (2022), Pan et al. (2022)

Before elaborating on the distinct cell types investigated for the management of tSCI (Table 1), it is important to acknowledge one of the largest challenges in the deployment of cell-based therapies, which is product consistency. A myriad of factors potentially influence the efficacy of cell products thereby affecting their therapeutic potential and ultimately patient outcomes. Some of these factors possibly introducing product heterogeneity include cell/product handling (such as various passage number, type of cell line, culture conditions, storage, shipment), cell delivery techniques (intravenous, intrathecal or intraparenchymal administration), cell dosing as well as the choice of the cell source (autologous versus allogeneic) along with their potential risk for immune-mediated graft rejection (Maartens et al., 2017; Hejrati et al., 2023). As such, translating cell-based therapies into clinical trials requires high quality standards. Therefore, Good Manufacturing Process (GMP) standards need to be established as early as possible, ideally in collaboration with regulatory bodies, in order to produce cell therapies, which are scalable, stable, effective, reproducible and safe with a highest chance of therapeutic potential (Lipsitz et al., 2016; Murray and Gibson, 2022).

The first human trial of OECs confirmed the safety of transplanting purified OECs into the spinal cord, at least until a three-year follow-up (Mackay-Sim et al., 2008). Of advantage, OECs can be harvested from autologous nasal mucosa thereby avoiding issues related to genetic incompatibility. However, a subsequent study, which transplanted mucosal tissue, rather than purified OECs, reported conflicting results, exposing implant-associated intramedullary spinal cord masses containing thick mucus-like material, which required resection (Dlouhy et al., 2014). In a subsequent development, results from a phase I trial indicated motor and sensory improvements along with the absence of relevant adverse events at one year after transplanting autologous OECs and olfactory nerve fibroblasts into the spinal cords of six patients with AIS grade A SCI (Tabakow et al., 2013). However, larger sample sizes and sensitive outcome parameters will be necessary to further confirm efficacy (Dlouhy et al., 2014).

Schwann cells (SCs) are crucial contributors to the inherent repair mechanisms of peripheral nerves. They can dedifferentiate, migrate, proliferate, express growth-promoting factors, and myelinate regenerating axons (Oudega and Xu, 2006). SCs secrete exosomes, which promote axonal regeneration both *in vitro* and *in vivo* (Ching et al., 2018) and can potentially enhance axonal function and neuronal sprouting, as well as reduce the formation of spinal cord cavitation. As shown in a recent study, SCs promote regeneration of axons, salvage motor function and reduce neuronal apoptosis following tSCI in rats (Pan et al., 2022). Recent studies show that transplanted SCs may also be neuroprotective by reducing the pro-inflammatory response after SCI (Pearse et al., 2018). A phase 1 open-label study (Gant et al., 2022) demonstrated feasibility and safety of SC-transplantation in humans with chronic cervical and thoracic AIS grade A-C SCI’s. Further trials are needed to evaluate the efficacy of SC transplantation for the treatment of tSCI.

Mesenchymal stem cells (MSCs) derived from umbilical cord, myeloid and adipose tissues as well as bone marrow-derived mesenchymal stem cells (BMSCs), were extensively investigated in rat models (Oliveri et al., 2014). MSCs can differentiate into myocytes, osteoblasts, chondrocytes and adipocytes (Ahuja et al., 2017). However, their beneficial role for the treatment of tSCI has been shown to lie in the modulation of the inflammatory response occurring during the second injury phase by exertion of immunomodulatory, anti-inflammatory, neurotrophic and angiogenetic factors (Lee et al., 2016). Moreover, they have demonstrated a favorable safety profile in a multitude of preclinical and clinical studies (Cofano et al., 2019). Yet, positive results from pre-clinical studies could not be reproduced in clinical studies. Some studies have shown slight improvements in motor function (Cofano et al., 2019), but the results were not statistically significant. The only published phase 3 clinical trial by Oh et al. is a non-randomized study (Oh et al., 2016). This trial involved a limited number of patients, with two out of 16 patients exhibiting motor improvement. Notably, these patients had incomplete injuries and underwent a standard rehabilitation program, raising the likelihood of spontaneous improvement (Kazim et al., 2021). In a recent placebo-controlled randomized trial, the clinical effectiveness of intramedullary administered BMSCs was assessed in 13 patients with acute complete SCI (Saini et al., 2022). Notably, only sensory function improved, with a mean ASIA score increase from 124 to 224 at 6 months, as opposed to controls with a consistent mean score of 115. No motor functional improvement was observed in any of the patients. Hence, the efficacy of MSC treatment in SCI remains irresolute.

Initial attempts with embryonic stem cells faced ethical concerns and supply limitations. A breakthrough in stem cell research overcame these challenges with the creation of induced pluripotent stem cells (iPSC), that can be derived from any somatic cell, including autologous sources (Takahashi and Yamanaka, 2006). Differentiated cells can be reprogrammed to an embryonic-like state by transfer of nuclear contents into oocytes or by fusion with embryonic stem (ES) cells. Designated as iPSCs, these reprogrammed cells exhibit the morphology and growth properties of ES cells, expressing ES cell marker genes (Takahashi and Yamanaka, 2006).

Neural stem cells (NSCs) have been discussed to be able to replace cells lost after SCI due to their ability to generate neurons and glial cells (Martin-Lopez et al., 2021). Animal models have shown increased locomotor rehabilitation after intrathecal application of neural stem cells. For example, Tai et al. (2021) found that SOX2 expression in NG2 glia, which are progenitor cells of oligodendrocytes, may induce neurogenesis. Several clinical trials have been launched to use neural stem cells at various levels of differentiation in humans (Martin-Lopez et al., 2021). Unfortunately, a rate-limiting step in the use of NSCs is that they cannot be harvested from autologous sources with their isolation depending on the provision of allogeneic donors. Therefore, the effectiveness of allogeneic CNS cell sources (such as NSCs) greatly depends on the harvest conditions, donor age as well as genetics (Hejrati et al., 2023).

However, recent achievements have allowed for the generation of NSCs from induced pluripotent stem cells (iPSCs), which then can be modulated to even more mature cells. The surrounding environment of the iPSCs influences their differentiation and may induce synapse formation similar to neurodevelopment (Takahashi and Yamanaka, 2006). Furthermore, iPSC-NSCs have the ability to enhance remyelination and axon regeneration, therefore leading to improved recovery of motor function in rats (Martin-Lopez et al., 2021), as evaluated by an array of different functional recovery assessments ranging from locomotor tests (like the Basso, Beattie, and Bresnahan test) to subjective evaluations or operator experience.

Biotechnology-led trials include the recently terminated investigation of a human fetal neural stem cell product (HuCNS-SCs; StemCells, Inc.) and an ongoing trial by Neuralstem of transplanted NSI-566, which are stem cells derived from the human fetal spinal cord (Ahuja et al., 2017). Regarding this, a phase I/II study concerning the safety and preliminary efficacy, was successful with intramedullary spinal cord transplantation proved safe and feasible using a manual injection technique (Levi et al., 2018), however, without early evidence of meaningful motor recovery. At six-year follow-up, the patient cohort receiving NSC transplantation following tSCI displayed no evidence of tumor formation or malformation in the lesion area (Curt et al., 2020). This observation indicates the safety and feasibility of cell transplantation in both the short and long term. Presently, only a few ongoing research initiatives are exploring the utilization of NSCs in human SCI. The “Safety Study of Human Spinal Cord-derived Neural Stem Cell Transplantation for the Treatment of Chronic SCI” focuses on patients with AIS grade A SCI’s. The study assessed the impact of Human Spinal Cord-derived Neural Stem Cell (HSSC) transplantation on AIS level, ASIA motor and sensory scores, bowel and bladder function, pain, evoked sensory and motor potentials, and electromyogram (EMG). The results are pending publication (NCT01772810).

Oligodendrocyte progenitor cells (OPCs) can be derived from a singular line of self-renewing human embryonic stem cells (hESCs) or from neural stem cells and demonstrate a propensity to differentiate into myelinating oligodendrocytes. In preclinical studies, OPCs have demonstrated the capability to produce neurotrophic factors, migrate within the spinal cord parenchyma, promote vascularization, and initiate remyelination of denuded axons (Zhang et al., 2006). These functionalities align with critical roles attributed to oligodendrocyte progenitor cells (OPCs) (Zhang et al., 2006). A phase I/II study on the safety of intraparenchymally injected OPCs (termed LCTOPC1) administered at a single time point between 21- and 42-days post injury, showed no relevant side effects and was well tolerated in patients with AIS grades A-B tSCI (Fessler et al., 2022). The safety and neurological function data support further investigation to determine the efficacy of LCTOPC1 for the treatment of tSCI (NCT023021579).

Hematopoietic Stem Cells (HSCs) can be harvested from adult bone marrow, the placenta, and cord blood. They can be differentiated into cells from all hematopoietic systems, namely myeloid cells and lymphocytes. Implanted into the SCI microenvironment, HSCs exert their therapeutic effects by differentiating into macrophages and different types of blood cells, as well as releasing a number of cytokines such as granulocyte colony-stimulating factor (*G*-*CSF*) and stem cell factor (SCF) (Szymoniuk et al., 2022). Within the SCI microenvironment, HSCs demonstrate the ability to differentiate into astrocytes, neuroprotective glia, and oligodendrocytes, as well as suppressing astrogliosis and thus glial scar formation (Frolov and Bryukhovetskiy, 2012; Xiong et al., 2017). Interestingly differentiated cells show positivity for markers specific for motor neurons (Moghaddam et al., 2017), underlining the potential of HSCs to differentiate into motor neuron-like cells. Numerous animal studies of tSCI have reported functional neurologic recovery following transplantation of HSCs (Koda et al., 2005; Xiong et al., 2017). Clinical studies assessing the efficacy of HSCs for the treatment of tSCI have also been conducted (Bryukhovetskiy and Bryukhovetskiy, 2015; Zakerinia et al., 2018). In a study by Deda et al., nine patients were transplanted HSCs following AIS grade A SCI. All nine patients with chronic SCI showed improved motor and sensory recovery three weeks after surgery, improving to AIS grade B or C (Deda et al., 2008). Conversely, a study involving 202 patients with tSCIs demonstrated an improvement in quality of life and motor recovery in only 15 patients as indicated by the ASIA motor score and functional independence measure (FIM) (Bryukhovetskiy and Bryukhovetskiy, 2015).

Bone marrow mononuclear cells (BMMNCs) contain a mixture of different cells including MSCs, HSCs, myeloid, lymphoid, and non-hematopoietic precursor cells (Garcia-Ayuso et al., 2022), and were evaluated for sub-acute and chronic SCI patients (Sharma et al., 2020). The intrathecally administered mixture, obtained from bone marrow (aspirated from the anterior iliac crest), demonstrated improvement in motor, sensory, and bladder function, indicated by FIM (functional independence measure) outcomes and the Walking Index for SCI (WISCI), without evidence of severe complications (Sharma et al., 2020). Due to the combined effects of the mentioned stem cell types and the findings from the studies mentioned earlier, BMMNCs represent a repromising therapeutic avenue for future research.

### Adeno-associated virus vectors and gene therapy

The basic idea of gene therapy involves introducing functional genes into cells that are either missing or defective, using vectors, which may involve viruses that have been modified to carry therapeutic genes of interest. Currently, the preferred vector for targeting a variety of nervous system conditions is the adeno-associated viral (AAV) vector because of its favorable safety profile and ability to target neurons effectively (Hudry and Vandenberghe, 2019). AAV vectors, which belong to the family of Parvoviridae, are multimeric proteins. The capsid provides protection from nucleases and immune sensing both inside and outside the cell and plays a crucial role in various aspects of its interaction with the host, including cell attachment and entry (Hudry and Vandenberghe, 2019). Once inside the target cell, AAV vectors integrate therapeutic genes into the host cell’s genome. Unlike other gene therapy vectors, such as lentiviral vectors and other non-AAV vectors, AAV vectors have attracted particular interest in the field of CNS research due to their safety and ability to sustain long-term transgene expression (Stepankova et al., 2021). One of the best studied methods for recreating a developmental environment within the CNS and the spinal cord involves inducing the expression of neurotrophic factors. One such factor is Ciliary neurotrophic factor (CNTF), which belongs to the interleukin 6 (IL-6) cytokine family (Richardson, 1994). CNTF has the ability to promote neuronal survival and facilitate long-distance regeneration of damaged neurites in various regions of the adult CNS (Richardson, 1994). In a recent study involving rats (Ruitenberg et al., 2004), AAV vectors were used to integrate brain-derived neurotrophic factor (BDNF) with stereotaxic administration of recombinant AAV into lesioned neurons in the red nucleus (RN), immediately after cervical axotomy, transducing neurons in the RN for up to 18 months post-injury and the reversal of neuronal atrophy during both the acute and chronic stages post-injury. Another preclinical study of tSCI in a rat T9 contusion model applied AAV vectors containing the acidic fibroblast growth factor (aFGF) as a potent neurotrophic factor for integration into neurons and astrocytes in the injured spinal cord (Huang et al., 2011). Subsequently, intracellular aFGF gets translocated into the nucleus, where it acts as a transcription factor to trigger DNA and protein expression, increasing the intrinsic activity of neurons, which is essential for neuronal regrowth and regeneration (Kadoya et al., 2009) leading to enhanced motor recovery, as measured by the BBB test (Huang et al., 2011). In another rat model of T10 thoracic tSCI, the administration of AAV vectors holding genes coding for human disintegrin and metalloproteinase with thrombospondin motifs-4 (ADAMTS4), into the injured spinal cord, successfully transduced spinal cord astrocytes and facilitated degradation of chondroitin sulfate proteoglycans (CSPGs). This process promoted sprouting of hindlimb corticospinal tract axons and led to an increase in the density of serotonergic fibers caudal to the injury site. Notably, these findings were observed in a contusive experimental model that closely resembles human tSCI (Griffin et al., 2020).

Although the therapeutic efficacy appears promising in preclinical rodent models, transition to clinical trials currently poses challenges and relevant risks. One of them being the potential risk of immune responses to humans, who may harbor neutralizing antibodies against viral capsids potentially blocking the therapeutic effect (Stepankova et al., 2021). Another issue to consider in AVV vectors and gene therapies in general, is the risk of tumorigenesis. AAV does not typically integrate into the host genome but remains in a non-replicating episomal state within the target cell after transduction (Schnepp et al., 2005). As a result, its expression is gradually lost in dividing cell populations, as there is no mechanism to actively duplicate the transgene during mitosis. However, there is a low risk of infrequent integration of the AAV transgene into the host genome (Donsante et al., 2007). This represents a potential risk for insertional mutagenesis, although such safety concerns have not yet been reported in human patients. Nevertheless, systemic delivery of AVV vectors has been associated with an increased incidence of hepatocellular carcinoma (HCC) in newborn mice between 43 and 52 weeks post-injection (Donsante et al., 2007). A recent study conducted in piglets and non-human primates (NHPs) revealed that administration of a high dose of a specific intravenously injected AAV vector variant resulted in severe toxicity, leading to death or requiring euthanasia just days after vector administration (Hinderer et al., 2018). Toxicological observations included elevated levels of transaminases, degeneration of dorsal root ganglia, impaired ambulation, proprioceptive deficits, and ataxia in piglets (Hinderer et al., 2018). AAV gene therapies represent interesting biological agents, and the clinical understanding of this platform is still in its early stages. Additionally, host responses to AAV therapies can be unpredictable, varying based on factors such as disease state, route and site of administration, genetic predispositions, and other idiosyncratic variables (Stepankova et al., 2021).

The astroglial response represents an adaptive mechanism following injury while providing a cell population, which theoretically can be harnessed for the use of direct reprogramming into desirable cell types aimed at promoting neuroregeneration. *In vivo* direct reprogramming is a concept which utilizes viral vectors to deliver genetic constructs into host cells thereby potentially changing differentiation profiles. Notably, reports have shown *in vivo* conversion of NG2+ or GFAP-positive astroglial cells into neurons following delivery of gene constructs including NeuroD1, Sox2 or Asc11 following injuries to the CNS (Guo et al., 2014; Su et al., 2014; Chen et al., 2020). While *in vivo* direct reprogramming strategies offer the unique advantage of utilizing endogenous cell pools, thereby avoiding potential risks associated with cell transplantation, several obstacles need to be addressed. As outlined above, safety concerns related to viral delivery need to be considered. Second, specificity of promoters need to be optimized in order to achieve appropriate targeting of genes of interest into host glial cells. Finally, the timing of *in vivo* direct reprogramming needs to be considered. While during the acute phase of injury, the astroglial border exhibits protective properties by restricting the damage and influx of inflammatory cells and cytokines from spreading (Herrmann et al., 2008), the chronic phase might pose a more suitable time window for *in vivo* direct reprogramming.

Another challenge includes immune-mediated graft rejection, where the host’s immune system tracks transplanted cells and mounts an attack. This rejection involves various immune mechanisms, including antibody and T-cell responses, as well as complement activation (Moreau et al., 2013). To address immune-mediated graft rejection in tSCI treatments, several strategies are being explored, which include using cells from genetically matched donors. However, finding suitable donors presents a significant challenge due to genetic diversity (Vazirabad et al., 2019). Another strategy involves immune tolerance induction protocols which can train the host’s immune system to accept transplanted cells as self. These protocols involve administering donor-derived cells and tissues to the recipient before transplantation (Wang et al., 2020). Finally, genetically modifying transplanted cells, for example using the CRISPR-Cas9 gene editing technique is a way to knock out genes that are involved in immune recognition and activation pathways, (such as major histocompatibility complex (MHC) class I and II genes) thereby potentially mitigating the risk for immunogenicity (Wang et al., 2020).

### Biomaterials

Neuroregeneration is often impeded by post-injury cystic cavities lacking the necessary support for cell migration and axon growth (Tsintou et al., 2015). Biomaterials offer a promising solution by filling posttraumatic cavitation defects and providing a supporting structural architecture of the extracellular matrix for growing cells and tissues (Mothe et al., 2013). These materials can be engineered to biodegrade, release growth factors or scar-degrading enzymes and even be seeded with stem cells for enhanced engraftment (Salewski et al., 2015). Several biomaterials, such as HAMC (Mothe et al., 2013), QL61 (Zweckberger et al., 2016), and fibrinogen (Lu et al., 2012), have shown effectiveness in animal models of SCI across acute to chronic phases of tSCI. InVivo Therapeutics has engineered a Neuro-Spinal Scaffold (NSS), comprising a biodegradable, biocompatible, and highly porous polymer (Poly-lactic-co-glycolic acid-Poly-L-lysine). Preclinical studies have demonstrated the NSS’s capacity to induce appositional healing, preserving spinal cord tissue, mitigate post-traumatic cyst formation, lowering spinal cord tissue pressure, and fostering neural regeneration in patients with acute AIS grade A thoracic SCI (Layer et al., 2017). The most recent 24-month follow-up data from the INSPIRE trial (Kim et al., 2022), a prospective, single-arm, multicenter study, revealed no unanticipated or serious adverse device effects, following NSS-transplantation into the intramedullary spinal cord contusion cavity. Anticipated in 2028, findings from the two-arm INSPIRE 2.0 study will assess, whether the NSS promotes functional recovery in complete T2-T12 SCIs, compared to the standard of care involving open spine surgery and not receiving the scaffold (NCT03762655).

Another exciting area are nanoparticles, which are becoming increasingly prominent in experimental models for SCI treatment, displaying diverse compositions including polymers, metals, metal oxides, silica, and biological molecules (Sun et al., 2020; Jeong et al., 2021). In a previous investigation, a biodegradable Mg/Al Lactate Dehydrogenase (LDH) was employed as a novel strategy for immune microenvironment modulation and neural regeneration. A recent study by Cheng et al. revealed that both LDH and LDH-NT3 demonstrated the capacity to enhance the microenvironment, expediting neural stem cell (NSC) migration, neural differentiation and L-Ca2+ channel activation (Cheng et al., 2021). Moreover, LDH-NT3 exhibited notable efficacy in modulating synaptic transmission and neuron–neuron synaptic communication (Cheng et al., 2021; Hu et al., 2023). The improved microenvironment established by LDH/LDH-NT3 positively regulated neural precursor cell synthesis, axonogenesis, and ion channel action-involved signaling pathways, fostering neuronal regeneration and the reconstruction of neural circuits post-SCI (Cheng et al., 2021). Graphene and graphene-based materials, known for their good electrical conductivity, hold potential for leveraging nerve electrical signals in spinal cord tissue to promote axon regeneration (Wang et al., 2022).

Biomaterials have emerged as effective carriers for cells and drugs. This combinatorial approach offers advantages, such as spatially and temporally controlled drug delivery to the injury site, while providing structural and guidance cues for endogenous and transplanted cells (Feng et al., 2023). For example, HA hydrogels have been shown to improve drug and stem cell delivery to the injury site while effectively enhancing cell survival and integration into host tissues (Khaing and Seidlits, 2015). In combination with adult brain-derived neural stem/progenitor cells (NSPCs) and recombinant rat platelet-derived growth factor-A (rPDGF-A), this method fosters oligodendrocyte differentiation, boosts graft survival, diminishes cavitation, and enhances behavioral outcomes (Mothe et al., 2013; Khaing and Seidlits, 2015). Another delivery approach involves the use of collagen, which is produced by fibroblasts and represents the main component of the extracellular matrix. It provides tensile strength for tissue growth and stimulates wound healing at the site of spinal injury (Feng et al., 2023). For targeted delivery of drugs and cells to SCI site, collagen can be engineered into diverse scaffold formats, including sponges, hydrogels, and guide catheters. In a recent study (Breen et al., 2017), a blend of neurotrophic factor (NT-3) and collagen hydrogel was administered via injection into a hemisected tSCI rat model. Results demonstrated enhanced neuronal and axonal growth, and a reduced inflammatory response and glial scar formation. Another novel vehicle for the implantation of stem cells, drugs, and cytokines are fibrin-based hydrogels. A 3D hierarchically arranged fibrin hydrogel (AFG) has been generated to mimic the natural spinal cord tissue environment, facilitating targeted host cell invasion, vascular system reconstruction, and axonal regeneration in rats with tSCI (Yao et al., 2018). Promoting differentiation of human endometrial stem cells into oligodendrocyte progenitors through upregulation of miR-219 expression and the use of fibrin hydrogels as a delivery scaffold for delivery of these cells to the SCI site significantly improved motor function recovery in SCI rats (Jalali Monfared et al., 2019).

Conclusively, biomaterials demonstrate biological properties facilitating nerve regeneration, while enhancing the efficacy of drug and cell delivery to the injury site when applied in a combinatorial fashion.

## Neuromodulation

Neuromodulation approaches try to activate electrical circuits of the remaining neurons in and around the lesion thereby evoking motor function. Two devices have been mostly studied, brain-spine interfaces (BSIs) and epidural spinal cord stimulation (ESCS).

The latter has been investigated for SCI since 2011. It was developed in the 1960s to treat chronic pain, but by chance and over time, the ability to evoke motor activity in individuals with SCI’s was detected (Mansour et al., 2022). The hypothesis behind ESCS for the treatment of SCI is that afferents are electrically stimulated, which can thereby, via interneurons and latent residual supraspinal translesional connections, activate locomotor centers and thus initiate movement (Hachmann et al., 2021). The frequency of the electrical impulse determines the locomotor action, e.g., standing vs. stepping. Its utility is being discussed for the recovery of sensory and autonomic functions (Ahuja et al., 2017). The usual neurosurgical approach is the implantation of an electrical grid onto the dorsal column of the spine via a laminotomy. The electrical grid can stimulate, mono-, bi- or multipolar and is operated by an implantable pulse generator. ESCS can be combined with physical rehabilitation, leading to significant improvement of motor function, and in some participants, including patients with AIS grade A sensorimotor complete SCI’s, even the ability to voluntary induce movement (Hachmann et al., 2021). Interestingly, in some patients, even when the ESCS device was switched off, voluntary movement was observed. Moreover, autonomous functions have been reported to regenerate as well as a secondary positive effect of ESCS. Stimulus frequency needs to be individualized in order to minimize adverse events such as clonic bursts. Other groups, Rowald et al. (2022), have investigated targeted nerve root stimulation to enable walking and generate a more natural gait pattern. The authors found improved coordination and better motor performance using their dynamic targeted stimulation approach compared to continuous stimulation. Implantation of an ESCS, even years after injury, holds the opportunity for improvement of functional outcomes in patients with chronic SCI (Ahuja et al., 2017). We hope that these early results will be reproduced in larger studies that also include cost–benefit analyses (Stienen and Ha, 2022).

Brain-Spine interfaces (BSI) aim at restoring the communication between the motor cortex and the spinal cord. Lorach et al. (2023) recently described a case where a 38-year-old participant with an incomplete SCI with an implanted BSI regained capability of walking, standing and climbing stairs (Lorach et al., 2023). BSI is suggested to induce even more natural gait patterns, establishing a link between the cortex and the analogue modulation of the epidural electrical stimulation, compared to targeted epidural electrical stimulation. The participant was able to walk, climb stairs and walk on natural uneven terrain with the device switched on (Sandrow-Feinberg and Houlé, 2015). The study group intends to test the device on patients with complete lesions. In general, these neuromodulative devices in combination with rehabilitation were shown to result in improved motor outcomes compared to rehabilitation only (Hachmann et al., 2021; Lorach et al., 2023).

## Conclusion

The consequences of tSCIs may significantly influence the physical and mental well-being of affected individuals. In the acute emergency setting, where patients present with severe neurological deficits, it is pivotal to deliver timely and specialized treatment. Following an initial evaluation, which includes a thorough and standardized neurological assessment and imaging diagnosis, individuals identified with tSCIs need to be considered for urgent surgical decompression and stabilization as soon as reasonably feasible, ideally within 24 h. For both patients deemed suitable for surgery and those treated non-surgically, medical management, particularly adequate hemodynamic therapy, can potentially positively influence neurologic recovery. Even minor improvements in outcome measures may translate into increased independence for self-care and reduced health-care costs. This circumstance has precipitated notable interest in both preclinical and clinical investigations concerning the management of tSCI. Despite numerous preclinical data demonstrating inefficacy upon translation into human subjects, the exploration of neuropharmacological, cell-based, and neuromodulatory strategies continues as a promising avenue for future investigations. While the field of tSCI has seen notable progress in recent years, the trajectory of future advancements is likely to involve the adoption of combinatory approaches aimed at synergistically amplifying functional outcomes for individuals with tSCI.

## Author contributions

GF: Conceptualization, Investigation, Methodology, Writing – review & editing, Data curation, Formal analysis, Visualization, Writing – original draft. LB: Conceptualization, Data curation, Formal analysis, Investigation, Methodology, Writing – original draft, Writing – review & editing. MS: Writing – review & editing. AC: Writing – review & editing. MF: Writing – review & editing. NH: Conceptualization, Investigation, Methodology, Project administration, Supervision, Writing – review & editing.
